# Docetaxel, Cisplatin and Fluorouracil (DCF) Regimen Compared with Non-Taxane-Containing Palliative Chemotherapy for Gastric Carcinoma: A Systematic Review and Meta-Analysis

**DOI:** 10.1371/journal.pone.0060320

**Published:** 2013-04-04

**Authors:** Xiao-Long Chen, Xin-Zu Chen, Chen Yang, Yan-Biao Liao, He Li, Li Wang, Kun Yang, Ka Li, Jian-Kun Hu, Bo Zhang, Zhi-Xin Chen, Jia-Ping Chen, Zong-Guang Zhou

**Affiliations:** 1 Department of Gastrointestinal Surgery, West China Hospital, Sichuan University, Chengdu, Sichuan, China; 2 Faculty of Medicine, West China School of Medicine, Sichuan University, Chengdu, Sichuan, China; 3 Chinese Cochrane Center, West China Hospital, Sichuan University, Chengdu, Sichuan, China; Vanderbilt University Medical Center, United States of America

## Abstract

**Background:**

Gastric carcinoma (GC) is one of the highest cancer-mortality diseases with a high incidence rate in Asia. For surgically unfit but medically fit patients, palliative chemotherapy is the main treatment. The chemotherapy regimen of docetaxel, cisplatin and 5-fluorouracil (DCF) has been used to treat the advanced stage or metastatic GC. It is necessary to compare effectiveness and toxicities of DCF regimen with non-taxane-containing palliative chemotherapy for GC.

**Methods:**

PubMed, EmBase, Cochrane Central Register of Controlled Trials and China National Knowledge Infrastructure databases were searched to select relative randomized controlled trials (RCTs) comparing DCF to non-taxane-containing chemotherapy for patients with palliatively resected, unresectable, recurrent or metastatic GC. Primary outcome measures were 1-year and 2-year overall survival (OS) rates. Secondary outcome measures were median survival time (MST), median time to progression (TTP), response rate and toxicities.

**Results:**

Twelve RCTs were eligible and 1089 patients were analyzed totally (549 in DCF and 540 in control). DCF regimen increased partial response rate (38.8% vs 27.9%, p = 0.0003) and reduced progressive disease rate (18.9% vs 33.3%, p = 0.0005) compared to control regimen. Significant improvement of 2-year OS rate was found in DCF regimen (RR = 2.03, p = 0.006), but not of 1-year OS rate (RR = 1.22, p = 0.08). MST was significantly prolonged by DCF regimen (p = 0.039), but not median TTP (p = 0.054). Both 1-year OS rate and median TTP had a trend of prolongation by DCF regimen. Chemotherapy-related mortality was comparable (RR = 1.23, p = 0.49) in both regimens. In grade I-IV toxicities, DCF regimen showed a major raise of febrile neutropenia (RR = 2.33, p<0.0001) and minor raises of leucopenia (RR = 1.25, p<0.00001), neutropenia (RR = 1.19, p<0.00001), and diarrhea (RR = 1.59, p<0.00001), while in other toxicities there were no significant differences.

**Conclusion:**

DCF regimen has better response than non-taxane containing regimen and could potentially improve the survival outcomes. The chemotherapy-related toxicity of DCF regimen is acceptable to some extent.

## Introduction

Gastric carcinoma is one of the highest cancer-mortality diseases [1]–[3] with a high incidence rate in Asia [4]. A lot of patients are diagnosed at advanced even end stage carcinoma, indicating poor outcomes [5]. For resectable diseases, surgery is considered as the mainstream treatment [6]–[9]. Adjuvant or neo-adjuvant chemotherapy has also been proven to benefit the survival rate in some studies and meta-analyses [10]–[14]. For surgically unfit but medically fit patients, palliative chemotherapy is the main treatment [6], [12], [15]–[18]. The regimens of cisplatin and 5-fluorouracil (CF) or epirubicin, cisplatin and 5-fluorouracil (ECF) have been used widely [19], and were often considered as the reference regimens for advanced gastric cancer [20]. Among new generation chemotherapy regimens, docetaxel, which is a semisynthetic taxane, promoting the assembly and stabilization of microtubules to inhibit the depolymerization [21], has been used more and more extensively with more potent effects [18], [22]–[25]. The chemotherapy based on docetaxel may be effective [26], because docetaxel lacks cross-resistance with other anti-tumor drugs [23]. The chemotherapy regimen of docetaxel, cisplatin and 5-fluorouracil (DCF) has been used to treat the advanced stage or metastatic gastric carcinoma with encouraging survival outcomes [27]–[33] and better quality of life [34], [35] in several studies. However, it was reported in some researches [36]–[38] that more toxicity, such as hematotoxicity, happened in DCF than in other regimens. Therefore, evaluation of benefits against the chemotherapy-related toxicities was needed. Present systematic review and meta-analysis were done to evaluate the survival outcomes and toxicities of DCF for palliatively resected, unresectable, recurrent or metastatic gastric carcinoma, compared with those of non-taxane-containing regimens.

## Methods

No protocol was registered.

### Search Strategy

We searched the electronic databases of PubMed (http://www.ncbi.nlm.nih.gov/sites/entrez/), EmBase (http://www.embase.com/home), Cochrane Central Register of Controlled Trials (http://ovidsp.tx.ovid.com/sp-3.4.1b/ovidweb.cgi), and China National Knowledge Infrastructure Database (http://acad.cnki.net/Kns55/brief/result.aspx?dbPrefix=CJFQ) up to July 31, 2011. The search strategy in PubMed was as follows, (“docetaxel”[Supplementary Concept] OR docetaxel[Text Word]) AND (“stomach neoplasms”[MeSH Terms] OR (“stomach”[All Fields] AND “neoplasms”[All Fields]) OR “stomach neoplasms”[All Fields] OR (“gastric”[All Fields] AND “cancer”[All Fields]) OR “gastric cancer”[All Fields]) AND (“humans”[MeSH Terms] AND (Clinical Trial[ptyp] OR Meta-Analysis[ptyp] OR Randomized Controlled Trial[ptyp] OR Review[ptyp]) AND English[lang]). The search strategy was also referred in other electronic databases.

### Inclusion and Exclusion Criteria

Only randomized controlled trials (RCTs) were eligible for inclusion. Included patients were diagnosed with palliatively resected, unresectable, recurrent or metastatic gastric carcinoma. Both, patients with previous surgery and without, were acceptable. DCF palliative chemotherapy could be administrated as the first-line regimen. If the control arm was blank or contaminated with taxane, the trials were excluded. Response, survival outcomes or toxicities were mandatory to be reported.

### Selection, Assessment and Data Extraction

Two independent reviewers (Chen XL, Yang C) read the title and abstract of every searched citation to select eligible studies for further assessment. Full text of potentially eligible citation was retrieved and determined for inclusion. Jadad scale described by Jadad, et al was used to assess the quality of RCTs [39]. Data was extracted independently by two reviewers mentioned above. Primary outcome measures included 1) 1-year and 2-year overall survival (OS) rates. Secondary outcome measures were 2) median survival time (MST), 3) time to progression (TTP), 4) response rate (WHO Criteria) [40] containing complete response (CR), partial response (PR), stable disease (SD), progressive disease (PD) and overall response rate (ORR), 5) toxicities (grade I–IV) and 6) chemotherapy-related death. ORR meant the combination of CR and PR. Study sample and regimen details were also extracted. Any disagreements in studies assessment and data collection were discussed and resolved by a third party (Hu JK, Chen XZ) as the referees.

### Statistical Analysis

The statistical analysis was performed by Reviewer Manager (RevMan) software, version 5.0 offered by The Cochrane Collaboration. The Mantel-Haenszel (M-H) test was used for comparison of dichotomous data and risk ratio (RR) or risk difference (RD) estimate. The 95% confidence interval (CI) of RR or RD was also calculated. A two-sided P value less than 0.05 was considered as a significant difference. Between-trials heterogeneity was evaluated by the Chi-square test; P value less than 0.1 was considered as significant heterogeneity [41]. Providing heterogeneity existed, random effect model was used for meta-analysis; otherwise fixed effect model was used.

## Results

### Literatures Search and Selection

**Figure 1:**
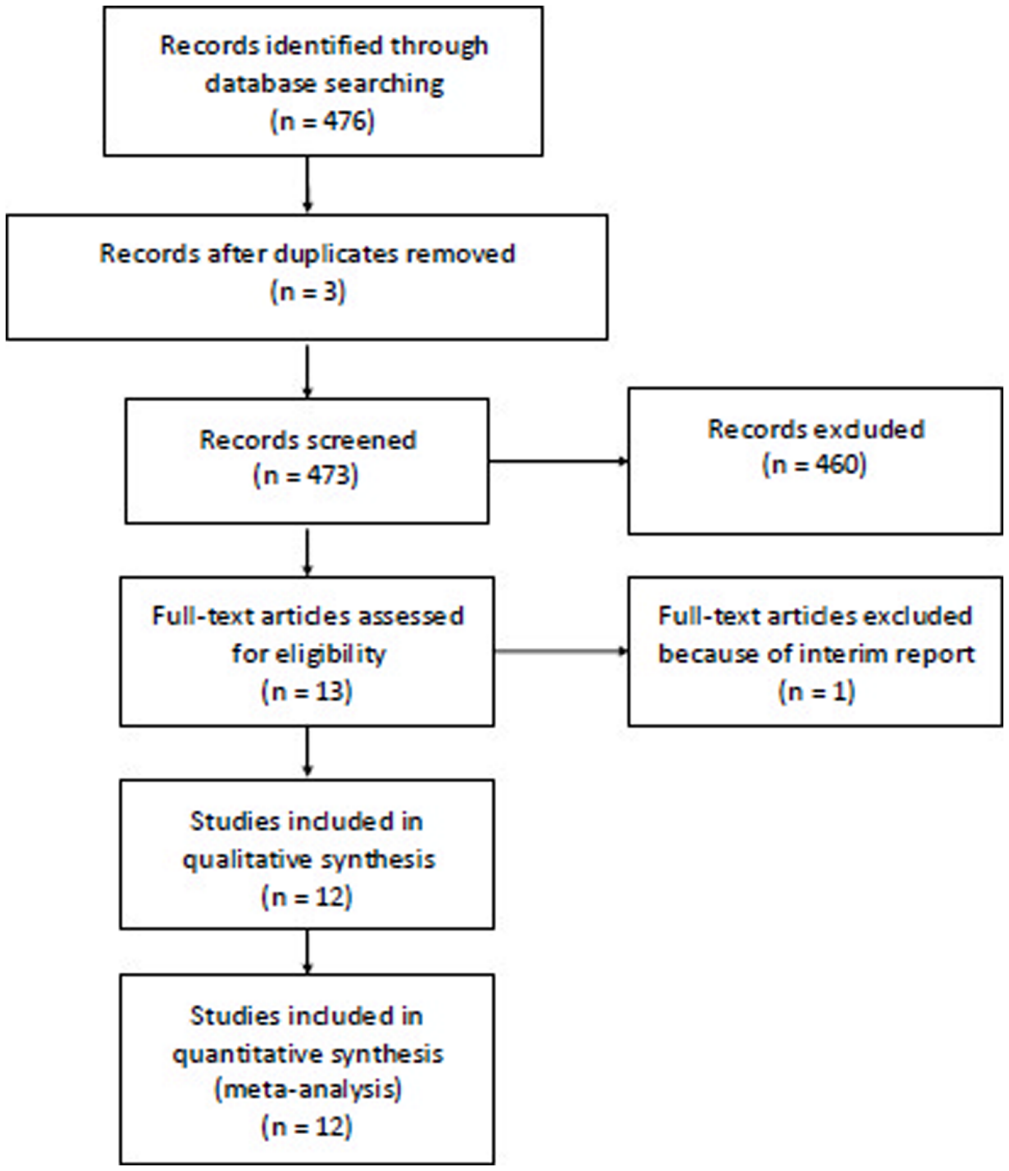
Literature search and selection procedure.

The literature search and selection procedure were shown in Figure 1. Totally, 12 RCTs were eligible for analysis and 1089 patients with palliatively resected, unresectable, recurrence or metastatic gastric cancer were involved (549 in DCF vs 540 in control) [35], [38], [42]–[51] (Table1). The sample size of individual RCT ranged from 36 to 445. There were no significant differences in the baselines between DCF and controlled group in these studies, as reported. In these studies, DCF were compared with cisplatin and fluorouracil (CF), epirubicin, cisplatin and fluorouracil (ECF), oxaliplatin and fluorouracil (FOLFOX4), etoposide and fluorouracil (EF) regimens.

**Table 1:** Summary information of included RCTs.

Studies	Demographic data	Intervention& control	Outcome measures	Jadad scores
**Chu JH, et al** [**42**]20061 center in China	40 patients with recurrence or metastaticgastric carcinoma chemotherapy-naïvewithin 1 month.	**DCF:** docetaxel 25 mg/m^2^, cisplatin 35 mg/m^2^, 5-FU750 mg/m^2^.3–4 cycles.**CF:** cisplatin 20 mg/m^2^, 5-FU 1000 mg/m2. 3–4 cycles.	ORR, MST, TTP andtoxicities	1
**Van Cutsem E, et al** [**43**] 200672 centers in 16 countries.	457 patients with metastaticor locally advanced/recurrentgastric cancer.12 patients without treatmentwere excluded.	**DCF**: docetaxel 75 mg/m^2^, cisplatin 75 mg/m^2^ and 5-FU750 mg/m^2^. Median6 (1–16) cycles. **CF**: cisplatin100 mg/m^2^ and 5-FU1000 mg/m^2^. Median 4 (1–12) cycles.	ORR,TTP, OS, toxicities and QOL	2
**Sadighi S, et al** [**35**]20061 center in Iran	86 patients with primary or recurrent gastric cancer(III–IV stage). 15 patients did not completethe questionnaires andwere excluded in QOL analyses.	**DCF**: docetaxel 60 mg/m^2^, cisplatin 60 mg/m^2^ and 5-FU750 mg/m^2^. 3–6 cycles**ECF**: epirubicin 60 mg/m^2^, cisplatin 60 mg/m^2^ and 5-FU750 mg/m^2^. 3–6cycles.	ORR and QOL	2
**Li XQ, et al** [**44**]20071 center in China	60 patients with stage IV gastric carcinoma.	**DCF**: docetaxel 25 mg/m^2^, cisplatin 6 mg/m^2^ and 5-FU200 mg/m^2^. 2 cycles.**CF**: cisplatin 6 mg/m^2^ and 5-FU 200 mg/m^2^. 2 cycles.	ORR, MST andtoxicities	1
**Roth AD, et al** [**38**]200713 centers in 4countries	121 patients with unresectablegastric cancer,metastatic or locally carcinoma. 2 patients withouttreatment were excluded.	**DCF**: docetaxel 85 mg/m^2^, cisplatin 75 mg/m^2^, and5-FU300 mg/m^2^. Median 4 cycles.**ECF**: epirubicin 50 mg/m^2^, cisplatin 60 mg/m^2^, 5-FU 200 mg/m^2^.Median 5.5 cycles.	ORR, OS, toxicitiesand QOL	2
**Wu GC, et al** [**45**]20081 center in China	58 patients with stage III–IV gastriccarcinoma received first orsecondary treatment.	**DCF:** docetaxel 75 mg/m^2^, cisplatin 75 mg/m^2^, 5-FU 750 mg/m^2^.2 cycles.**CF:** cisplatin 75 m/m^2^, 5-FU 750 mg/m^2^. 2 cycles	ORR, MST, andtoxicities	1
**Zhang FL, et al** [**46**]20081 center in China	50 chemotherapy-naive patients with localrecurrence or metastatic carcinoma.	**DCF:** docetaxel 75 mg/m^2^, cisplatin 25 mg/m^2^, 5-FU 500 mg/m^2^. More than2 cycles.**CF:** cisplatin 25 m/m^2^, 5-FU 500 mg/m^2^.More than 2 cycles.	ORR and Toxicities	2
**Hou AJ, et al** [**47**]20091 center in China	40 patients with stage IIIB–IV after gastrectomy orpalliative surgery. 4 patients could not be evaluated.	**DCF:** docetaxel 40 mg/m^2^, cisplatin 30 mg/m^2^ and 5-FU 200 mg/m^2^.More than 2 cycles.**ELF:** etoposide 120 mg/m^2^, 5-FU 500 mg/m^2^. More than ^2^ cycles.	ORR, OS, MST andQOL	3
**Zhao F, et al** [**48**]20091 center in China	31 gastric cancer patients in DCF arm and32 in FOLFOX4 arm with recurrenceafter radical gastrectomy or withoutsurgery because ofmetastasis.	**DCF:** docetaxel 75 mg/m^2^, cisplatin 20 mg/m^2^ and 5-FU 350 mg/m^2^. Median 3.1 cycles.**FOLFOX4:** oxaliplatin 100 mg/m^2^ and5-FU 400 mg/m^2^.Median 3.2 cycles.	ORR, TTP and MST	1
**Shen YC, et al** [**49**]20091 center in China.	48 chemotherapy-naive patients with late stage gastric carcinoma after noor palliative surgery.	**DCF:** docetaxel 35 mg/m^2^, cisplatin 6 mg/m^2^ and 5-FU250 mg/m^2^. 3–4 cycles**CF:** cisplatin 75 mg/m^2^ and 5-FU 1000 mg/m^2^. 3–4 cycles	ORR, TTP andtoxicities	1
**Liang B, et al** [**50**]20101 center in China	58 patients in DCF arm and control armwith advanced gastric cancer expectedto survive more than3 months.	**DCF:** docetaxel 75 mg/m^2^, cisplatin 75 mg/m^2^ and 5-FU 300 mg/m^2^.More than 2 cycles.**ECF:** epirubicin 50 mg/m^2^, cisplatin 60 mg/m^2^, 5-FU 200 mg/m^2^. Morethan 2 cycles.	ORR and toxicities	1
**Gao H, et al** [**51**]20101 center in China	64 patients with stage IIIB–IV gastric carcinoma.	**DCF:** docetaxel 60 mg/m^2^, cisplatin 25 mg/m^2^, 5-FU 1000 mg/m^2^. More than 2 cycles.**ECF:** epirubicin 50 mg/m^2^, cisplatin 25 mg/m^2^, 5-FU 1000 mg/m^2^. Morethan 2 cycles.	ORR, OS and QOL	3

**Abbreviations: DCF:** docetaxel, cisplatin and fluorouracil; **CF:** cisplatin and fluorouracil; **ECF:** epirubicin, cisplatin and fluorouracil; **FOLFOX4:** oxaliplatin and fluorouracil; **EF:** etoposide and fluorouracil; **FU:** fluorouracil; **ORR:** overall response rate; **TTP:** time to progression; **QOL:** quality of life; **MST**: median survival time; **OS:** overall survival.

### Response

**Figure 2:**
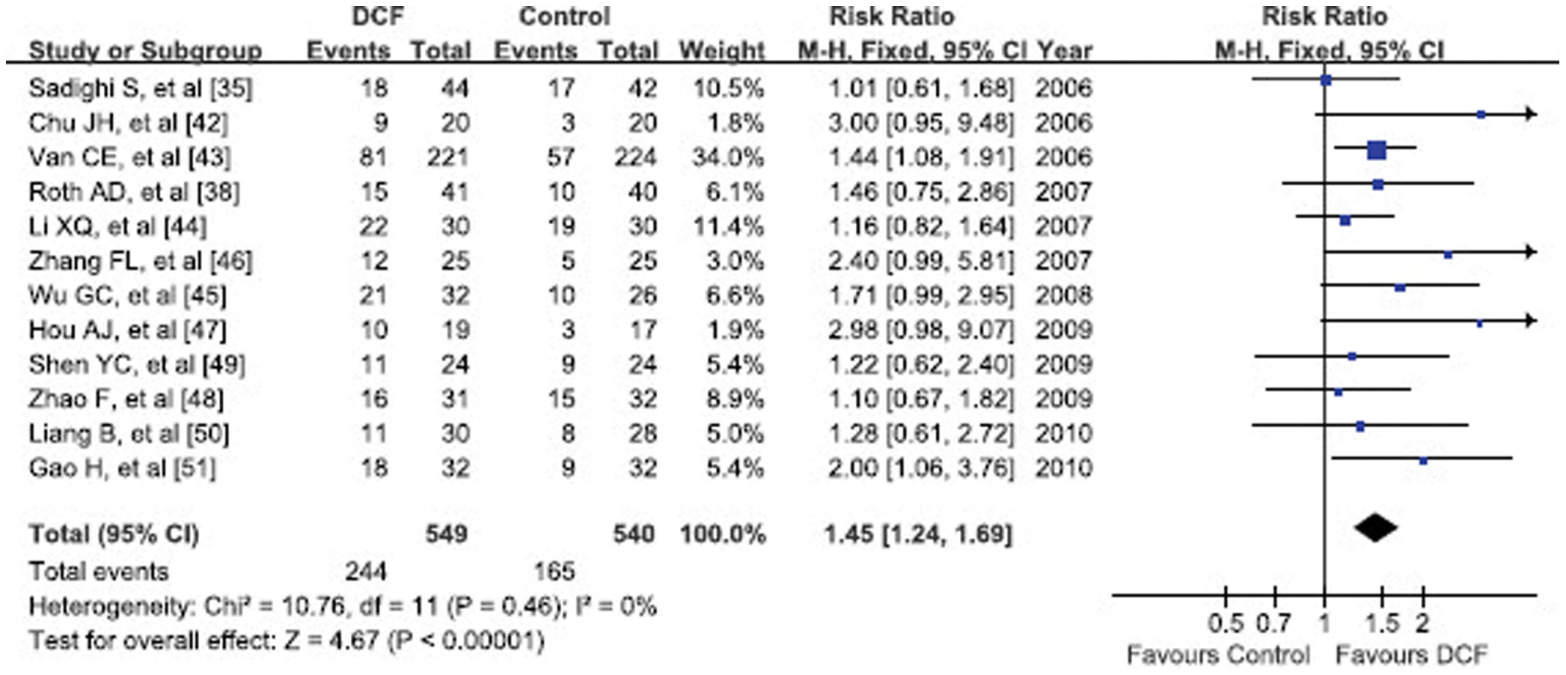
Forest plot of overall response rate.

Response rate was based on WHO Criteria [40]. The overall response rate (ORR) combined of CR and PR, was 44.4% (244/549) vs 30.6% (165/540) in DCF and non-taxane-containing regimens, respectively. Its meta-analysis showed significantly better ORR of DCF regimen (RR = 1.45, 95% CI 1.24–1.69, p<0.00001) (Figure 2). The subgroup meta-analysis results of CR, PR, SD and PD were listed in Table 2. CR and SD rates were not significantly different between two groups (Table 2). DCF regimen was able to noticeably increase PR rate (38.8% vs 27.9%, RR = 1.39, 95%CI 1.16–1.65, p = 0.0003), as well as reduce PD rate (18.9% vs 33.3%, RR = 0.65, 95%CI 0.51–0.83, p = 0.0005) (Forest plots not shown).

**Table 2:** Response comparison between DCF and non-taxane-containing chemotherapy.

Response	Study	DCF			Non-taxane-containing			PooledRR	95% CI	P value	Model	References
	counts	Events	Total	Accumulated	Events	Total	Accumulated					
				percentage			percentage					
CompleteResponse (CR)	11	25	505	5.0%	14	498	2.8%	1.69	0.91–3.14	0.10	Fixed	[38], [42]–[51]
PartialResponse (PR)	11	196	505	38.8%	139	498	27.9%	1.39	1.16–1.65	0.0003	Fixed	[38], [42]–[51]
StableDisease (SD)	9	122	444	27.5%	136	438	31.1%	0.88	0.72–1.08	0.23	Fixed	[43]–[51]
ProgressiveDisease (PD)	9	84	444	18.9%	146	438	33.3%	0.65	0.51–0.83	0.0005	Fixed	[43]–[51]

**Abbreviations: DCF:** docetaxel, cisplatin and fluorouracil; **RR:** risk ratio.

### Survival Outcomes

**Figure 3:**
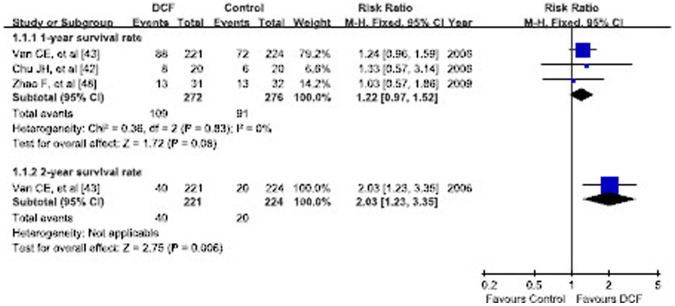
Forest plot of subgroup analysis of 1-year and 2-year overall survival rates.

Only 3 RCTs reported the 1-year OS rate and its details ranging from 40.0% to 41.9% in DCF and 30.0% to 40.0% in non-taxane-containing regimens [42], [43], [48]. The cumulative 1-year OS rate was 40.1% (109/272) in DCF and 33.0% (91/276) in non-taxane-containing regimens. Meta-analysis demonstrated no significant difference in 1-year OS rate (RR = 1.22, 95% CI 0.97–1.52, p = 0.08). However only one RCT analyzed 2-year OS rate showing significantly better 2-year OS rate in DCF regimen (18.0% vs 9.0%, RR = 2.03, p = 0.006) [38] (Figure 3).

**Figure 4:**
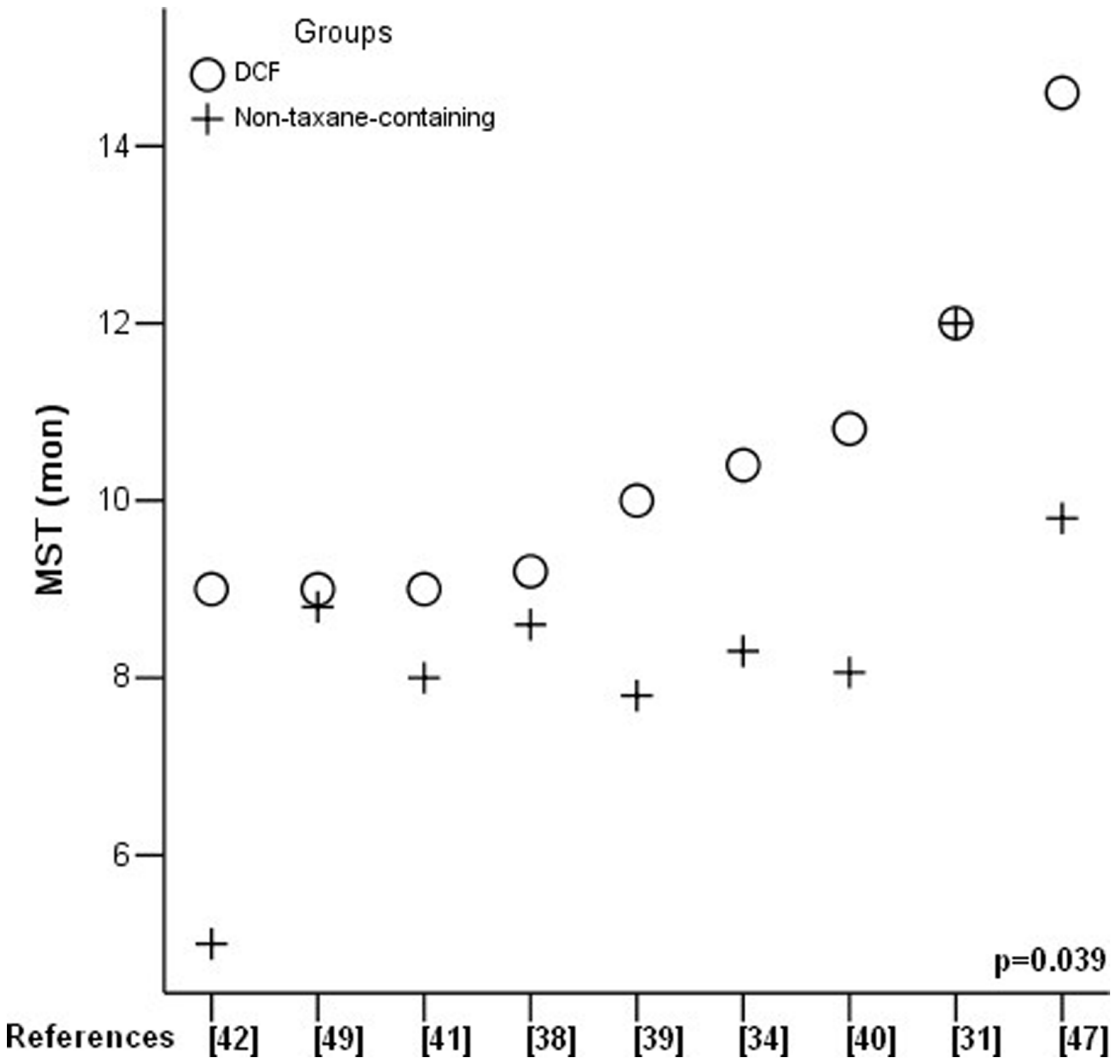
Median survival time (MST) comparison.

**Figure 5:**
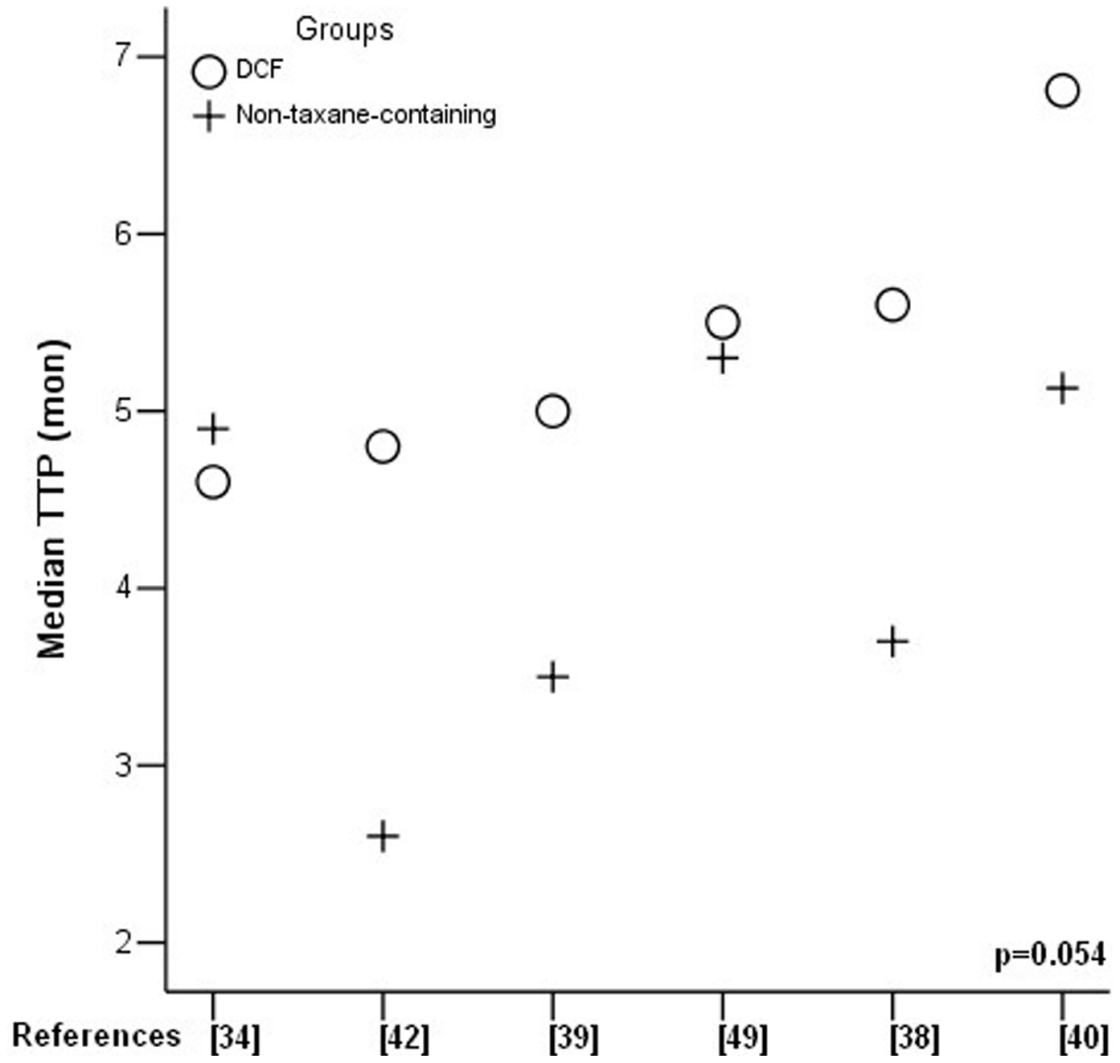
Median time-to-progression (TTP) comparison.

MST was reported in 9 RCTs and it ranged from 9.0 to 14.6 months in DCF and 5.0 to 12.0 months in non-taxane-containing regimens (Table 3). Six RCTs analyzed median TTP which ranged from 4.6 to 6.8 months in DCF and 2.6 to 5.5 months in non-taxane-containing regimens (Table 3). Comparison of MST and median TTP between two groups by one-way ANOVA test (SPSS 13.0) demonstrated significantly prolonged MST in DCF regimen (p = 0.039) (Figure 4). Median TTP showed a trend of prolongation in DCF regimen without reaching statistically significant difference (p = 0.054) (Figure 5).

**Table 3:** Details information of MST and TTP reported by some RCTs.

Studies	MST (range)		P value	Median TTP (range)		P value
	DCF	Non-taxane-containing		DCF	Non-taxane-containing	
Chu JH, et al [42]	10.0	7.8	–	5.0	3.5	–
Van CE, et al [43]	9.2 (8.4–10.6)	8.6 (7.2–9.5)	0.02	5.6 (4.9–5.9)	3.7 (3.4–4.5)	<0.001
Sadighi S, et al [35]	12.0 (7–17)	12.0 (8–14)	–	–	–	–
Li XQ, et al [44]	9.0	5.0	<0.05	4.8	2.6	<0.05
Roth AD, et al [38]	10.4 (8.3–12.0)	8.3 (7.2–13.0)	–	4.6 (3.5–5.6)	4.9 (3.2–6.1)	–
Wu GC, et al [45]	14.6	9.8	<0.05	–	–	–
Hou AJ, et al [47]	9.0 (2–18)	8.0 (2–18)	>0.05	–	–	–
Zhao F, et al [48]	9.0	8.8	>0.05	5.5	5.3	>0.05
Gao H, et al [51]	10.81 (8.42–13.20)	8.06 (6.46–9.67)	0.038	6.81 (5.52–8.11)	5.13 (4.18–6.07)	0.041

**Abbreviation: MST:** median survival time; **TTP:** time to progression; **DCF:** docetaxel, cisplatin and fluorouracil.

### Toxicities

In these studies, the toxicities were graded from I to IV according to WHO Criteria, National Cancer Institute-Common Terminology Criteria and National Cancer Institute of Canada Common Toxicity Criteria. These three different criteria are comparable. We compared grade I–IV and grade III–IV in both arms according to reports information (Table 4). The major grade I–IV toxicities (accumulated rate >5%) of DCF regimen were hematological toxicity including leucopenia (81.7%), neutropenia (84.1%), febrile neutropenia (27.3%), thrombocytopenia (24.7%), anemia (81.6%), and digestive systemic toxicity including diarrhea (58.9%), nausea/vomiting (59.2%), stomatitis (56.2%), anorexia (45.8%), constipation (26.3%), liver damage (7.3%). Other toxicities were neurological damage (33.7%), alopecia (73.5%), anaphylaxis (11.8%), infection (16.2%), fatigue (54.6%) and fluid retention (6.7%).

**Table 4:** Toxicities comparison between DCF and non-taxane-containing chemotherapy.

Toxicities	Study	DCF			Non-taxane-containing			Pooled	95% CI	P value	Model	References
	counts	Events	Total	Accumulated	Events	Total	Accumulated	RR/RD				
				percentage			percentage					
Leucopenia (I–IV)	6	282	345	81.7%	228	346	65.9%	RR = 1.25	1.15–1.35	<0.00001	Fixed	[42]–[44], [47], [49], [51]
III–IV	5	185	342	54.1%	97	342	28.4%	RR = 1.72	1.15–2.56	0.008	Random	[38], [43], [44], [47], [51]
Neutropenia (I–IV)	4	254	302	84.1%	215	303	71.0%	RR = 1.19	1.11–1.28	<0.00001	Fixed	[43], [47], [48], [51]
III–IV	4	185	302	61.3%	128	303	42.2%	RR = 1.46	1.28–1.66	<0.00001	Fixed	[43], [47], [48], [51]
Febrile neutropenia (I–IV)	2	69	253	27.3%	30	256	11.7%	RR = 2.33	1.57–3.44	<0.0001	Fixed	[43], [51]
III–IV	2	17	73	23.3%	7	72	9.7%	RR = 2.37	1.10–5.09	0.03	Fixed	[38], [51]
Thrombocytopenia (I–IV)	5	82	332	24.7%	102	334	30.5%	RR = 1.22	0.64–2.32	0.55	random	[43], [44], [47], [48], [51]
III–IV	6	19	373	5.1%	48	357	13.4%	RR = 0.60	0.33–1.08	0.09	Fixed	[38], [43], [44], [47], [48], [51]
Anemia (I–IV)	5	271	332	81.6%	265	334	79.3%	RR = 1.03	0.97–1.10	0.28	Fixed	[43], [44], [47], [48], [51]
III–IV	5	47	332	14.2%	61	334	18.3%	RR = 0.73	0.48–1.12	0.15	Fixed	[43], [44], [47], [48], [51]
Diarrhea (I–IV)	5	192	326	58.9%	122	330	37.0%	RR = 1.59	1.36–1.87	<0.00001	Fixed	[42]–[44], [48], [49]
III–IV	4	49	323	15.2%	20	326	6.1%	RR = 2.82	1.62–4.89	0.0002	Fixed	[38], [43], [44], [48]
Nausea/vomiting (I–IV)	7	223	377	59.2%	235	379	62.0%	RR = 0.96	0.86–1.07	0.42	Fixed	[42]–[44], [47]–[49], [51]
III–IV	6	49	374	13.1%	51	375	13.6%	RR = 0.97	0.68–1.38	0.85	Fixed	[38], [43], [44], [47], [48], [51]
Stomatitis (I–IV)	2	141	251	56.2%	142	254	55.9%	RR = 1.01	0.86–1.17	0.94	Fixed	[43], [44]
III–IV	3	49	292	16.8%	63	294	21.4%	RR = 0.79	0.57–1.09	0.15	Fixed	[38], [43], [44]
Anorexia (I–IV)	2	116	253	45.8%	116	256	45.3%	RR = 1.01	0.84–1.22	0.90	Fixed	[43], [51]
III–IV	2	28	253	11.1%	23	256	9.0%	RR = 1.23	0.73–2.08	0.44	Fixed	[43], [51]
Constipation (I–IV)	1	5	19	26.3%	2	17	11.8%	RR = 2.24	0.50–10.06	0.29	Fixed	[47]
III–IV	1	0	19	0.0%	0	17	0.0%	RD = 0.15	−0.10–0.40	0.25	Fixed	[47]
Liver damage (I–IV)	3	6	82	7.3%	7	81	8.6%	RR = 0.84	0.29–2.42	0.75	Fixed	[47], [48], [51]
III–IV	3	0	82	0.0%	0	81	0.0%	RD = 0.00	−0.04–0.04	1.00	Fixed	[47], [48], [51]
Neurological	2	85	252	33.7%	66	256	25.8%	RR = 0.41	0.02–9.09	0.58	Random	[43], [48]
damage(I–IV)												
III–IV	3	19	293	6.5%	8	296	2.7%	RR = 2.39	1.07–5.36	0.03	Fixed	[38], [43], [48]
Alopecia (I–IV)	2	36	49	73.5%	7	47	14.9%	RR = 3.75	1.01–13.84	0.05	Random	[44], [47]
III–IV	3	41	90	45.6%	8	87	9.2%	RR = 8.48	0.16–461.63	0.29	Random	[38], [44], [47]
Anaphylaxis (I–IV)	2	6	51	11.8%	1	49	2.0%	RR = 4.11	0.74–22.88	0.11	Fixed	[47], [51]
III–IV	2	0	51	0.0%	0	49	0.0%	RD = 0.00	−0.05–0.05	1.00	Fixed	[47], [51]
Infection (I–IV)	2	39	241	16.2%	28	244	11.5%	RR = 1.41	0.90–2.22	0.13	Fixed	[42], [43]
III–IV	1	28	221	12.7%	16	224	7.1%	RR = 1.77	0.99–3.19	0.06	Fixed	[43]
Fatigue (I–IV)	2	131	240	54.6%	108	241	44.8%	RR = 1.22	1.02–1.46	0.03	Fixed	[43], [47]
III–IV	2	41	240	17.1%	31	241	12.9%	RR = 1.34	0.87–2.06	0.18	Fixed	[43], [47]
Fluid retention (I–IV)	1	2	30	6.7%	0	30	0.0%	RR = 5.00	0.25–99.95	0.29	Fixed	[44]
III–IV	1	0	30	0.0%	0	30	0.0%	RD = 0.00	−0.06–0.06	1.00	Fixed	[44]

**Abbreviations: DCF:** docetaxel, cisplatin and fluorouracil; **RR:** risk ratio; **RD:** risk difference.

The meta-analysis results of these toxicities were listed in Table 3 (Forest plots not shown). There was significant increase of toxicities in DCF regimen in leucopenia (I–IV RR = 1.25, p<0.00001; III–IV RR = 1.72, p = 0.008), neutropenia (I–IV RR = 1.19, p<0.00001; III–IV RR = 1.46, p<0.00001), febrile neutropenia (I–IV RR = 2.33, p<0.0001; III–IV RR = 2.37, p = 0.03), diarrhea (I–IV RR = 1.59, p<0.00001; III–IV RR = 2.82, p = 0.0002), neurological damage (III–IV RR = 2.39, p = 0.03) and fatigue (I–IV RR = 1.22, p = 0.03). In grade I–IV toxicities, DCF regimen showed major raise of febrile neutropenia and minor raise of leucopenia, neutropenia, and diarrhea. Other toxicities such as thrombocytopenia, anemia, nausea/vomiting, stomatitis, anorexia, constipation, alopecia, neurological damage and infection appeared with no significant differences in both arms. Some toxicities including febrile neutropenia, liver damage, anaphylaxis and fluid retention were rare.

One RCT reported chemotherapy-related deaths within 30 days of the last infusion which were mainly caused by infection in both groups [43]. Four RCTs reported no chemotherapy-related mortality during the treatment [38], [45], [46], [50]. The accumulated chemotherapy-related mortality rates were 6.6% (23/349) in DCF and 5.5% (19/343) in non-taxane-containing regimens without significant difference (RR = 1.23, 95%CI 0.69–2.91, p = 0.49) (Forest plot not shown).

## Discussion

Although our meta-analysis did not show the significant difference of 1-year OS rate between DCF chemotherapy and non-taxane-containing regimens, we found that DCF arm was relatively better than control arms in cumulative 1-year OS rate, MST, median TTP and ORR. Only one study, which had a large sample size (445 patients), reported their 2-year OS rate (DCF 18% vs CF 9%) [43]. Many studies preferred to show the MST or OS time, TTP and especially ORR which represented the short survival outcomes. There was an interesting phenomenon in one study, in which DCF arm had shorter median TTP but longer MST than ECF arm [38]. According to our results, the difference of ORR was significant between two arms, with DCF having more benefits than control group in short time.

On the toxicity analysis, we found that DCF showed worse hematological toxicity, diarrhea, and fatigue. Many studies reported that these toxicities in both group could be accepted or controlled by granulocyte colony stimulating factors (G-CSF), antiemetic, vitamin B6 and drug discontinuance[38], [46], [49], [51]–[53]. Further, the total mortality of treatment, mainly caused by infection, was 6.6% in DCF arm and 5.5% in control arms, and showed no significant difference. Prophylactic antibiotics might be necessary for patients with severe leucopenia. Quality of life (QOL) after chemotherapy was of less focus than survival outcomes. Four RCTs demonstrated that DCF group did not have lower QOL. In fact, it even had obvious improvements in global QOL and Karnofsky performance status as well as prolonged the time to worsening of global health [35], [38], [43], [47]. Although causing more toxicities, DCF was a relatively safe and acceptable chemotherapy.

In every chemotherapy cycle for three or four weeks, the total dosage of docetaxel was from 105 mg/m^2^ to 300 mg/m^2^, cisplatin from 90 mg/m^2^ to 360 mg/m^2^, and 5-FU from 3000 mg/m^2^ to 15000 mg/m^2^ in these studies. The single dosage of docetaxel was noticeably lower in four studies from China (25 mg/m^2^ to 40 mg/m^2^) [42], [44], [47], [49], that was about one half of the dosage of three foreign studies (60 mg/m^2^ to 85 mg/m^2^) and five other Chinese studies (60 mg/m^2^ to 75 mg/m^2^) [35], [38], [43], [45], [46], [48], [50], [51] in which single dosage was similar to that recommended in National Comprehensive Cancer Network (NCCN) clinical practice guideline in oncology [54]. Similar phenomenon happened in cisplatin. Studies in China showed a noticeably lower single dosage of cisplatin (6 mg/m^2^ to 35 mg/m^2^) [35], [44], [46]–[49], [51] than foreign RCTs [35], [38], [43] (60 mg/m^2^ to 75 mg/m^2^) [45], [50] and NCCN recommendation [54]. From all these, we could see that there was a big difference in approach to dosage of DCF. The reason for this, we believe, was the individual distinctions of people in China and Western countries. Moreover, among Chinese RCTs, there was also a difference in the dosage of docetaxel and cisplatin. The question about which plan has better balance of survival outcomes and toxicities, a bigger single dosage with lower frequencies or a smaller single dosage with higher frequencies, needs further research.

A previous systematic review showed that there was significant difference in ORR but no significant difference in overall survival, when docetaxel was compared with non-taxane-containing regimens, which is in accord to our study [55]. However, in former review non-docetaxel-containing regimens might include both single and combined chemotherapy regiments. If docetaxel alone is compared with combined regimens, it might show relatively inferior efficacy of docetaxel. However, in our review, DCF was compared with other combined chemotherapy in all RCTs, which in our opinion seemed more reasonable.

In retrieved literatures of RCTs on DCF, we found that there were no studies from Japan. On one hand, it can be explained by the fact that we only retrieved the English or Chinese language articles. On the other hand, many studies [56]–[61] in Japan talked about the new generation chemotherapy drug S-1 alone or S-1 combined with other drugs, such as docetaxel, and reported promising effects in treating gastric carcinoma. However, they did not compare S-1 with DCF.

Further, we could see that DCF as palliative chemotherapy was reported in all RCTs. At present, adjuvant single or combined chemotherapy following radical surgeries included 5-FU, cisplatin, FOLFOX, capecitabine and S-1. Docetaxel was not the routine first line chemotherapy regimen. From NCCN introduction, DCF was recommended as first line chemotherapy for metastatic or locally advanced cancer [54]. It might raise the question whether or not DCF regimen could be used in postoperative adjuvant chemotherapy and have better survival outcomes than traditional adjuvant chemotherapy.

In our review, 9 out of 12 RCTs came from China, in which the population was small and follow-up time was short. Their aim was to evaluate the short term outcomes, ORR and toxicities. Only three RCTs including a foreign one reported the survival rate. This might influence the analysis in our review. More RCTs with longer follow-up time are needed.

### Conclusion

DCF regimen had better response than non-taxane containing regimen and could potentially improve the survival outcomes. The chemotherapy-related toxicity of DCF regimen is also acceptable to some extent. At the same time, more high-quality RCTs are needed to decide on the effectiveness of DCF regimen.
